# Results of an Online Community Needs Assessment for Psychoeducational Interventions Among Partners of Hereditary Breast Cancer Previvors and Survivors

**DOI:** 10.2196/jmir.1847

**Published:** 2012-01-18

**Authors:** Kenneth P Tercyak, Darren Mays, Tiffani A DeMarco, McKane E Sharff, Susan Friedman

**Affiliations:** ^1^Georgetown University Medical CenterWashington, DCUnited States; ^2^FORCE, Inc.Tampa, FLUnited States

**Keywords:** Breast cancer, hereditary cancer, social support, psychoeducation, psychosocial intervention

## Abstract

**Background:**

Spouses and partners (“partners”) of women at-risk for (“previvors”) and surviving with hereditary breast/ovarian cancer are a primary source of support within their families. Yet, little is known about partners’ needs for psychoeducational intervention to enhance their cancer risk knowledge, coping, and support role functioning.

**Objective:**

To determine the type and range of need for psychoeducational intervention among partners of hereditary breast cancer previving and surviving women, and to understand the potential role of the Internet and other communication channels in meeting that need.

**Methods:**

We conducted a secondary data analysis on partners’ needs that were originally assessed via an online community-based organization devoted to hereditary breast cancer. Partners’ demographic characteristics, need for psychoeducation, and likelihood of using various communication channels were assessed along with other constructs. Analyses examined commonly-occurring clusters of likely intervention use and by communication channel.

**Results:**

Partners (n =143) endorsed a moderately high level of need for psychoeducation and did so across multiple content areas (e.g., role functioning, decision making, communication, intimacy). Factor analysis identified three commonly-preferred communication channels: 1) self-help materials, 2) online interactions, and 3) interpersonal interactions. A cluster analysis among these factors identified three groups of partners based on their likelihood of psychoeducational intervention use (low [18%], moderate [55%], and high [27%] users). In a covariate-adjusted MANOVA, moderate and high intervention users reported significantly greater need for psychoeducation compared to low users (F_2,132_ = 9.15, P < .001).

**Conclusions:**

A majority of assessed partners perceived a need for psychoeducational interventions surrounding hereditary breast cancer risk. Internet-based, interactive resources may be an efficient mechanism to reach large numbers of partners with tailored content. Research is warranted to inform the design and deployment of these resources to ensure quality and high impact, and ultimately to examine ways to integrate these resources into clinical care.

## Introduction

Every year in the United States, breast cancer is diagnosed in over 200,000 women [1]: internationally, it is the second most commonly diagnosed malignancy and the leading cause of cancer-related death among women [2]. Of these cases, approximately 5%–10% are hereditary [3]. Most cases of hereditary breast cancer are attributable to germline mutations in one of two major breast cancer-predisposing genes, *BRCA1* or *BRCA2* (*BRCA1/2*) [4,5]. Women with a *BRCA1/2* mutation face up to an 85% lifetime risk for breast cancer and up to a 65% lifetime risk for ovarian cancer [4,5]. Moreover, these cancers are often diagnosed in women at younger ages than average [6]. Importantly, when a *BRCA1/2* mutation is identified in an individual, there is a 50% chance that first-degree relatives (eg, male and female children and siblings) have also inherited the mutation and may therefore face increased risks for cancer [5]. For women with a known *BRCA1/2* mutation, breast cancer screening consists of mammography and breast magnetic resonance imaging starting at age 25 years [7,8]. Breast cancer risk-reduction options include chemoprevention, prophylactic mastectomy, and prophylactic oophorectomy, or a combination of these [7,8]. Prophylactic oophorectomy is recommended after childbearing is completed to reduce mortality from ovarian cancer. Given all of these considerations, the presence of hereditary cancer confronts families with many complex, emotionally charged decisions, and increased awareness of familial cancer susceptibility brings about a lifelong impact [9-11].

Genetic counseling and testing for *BRCA1/2* mutations is a well-established component of the identification and management of hereditary breast and ovarian cancer syndrome among those who are at risk [12,13]. Though cancer care providers (eg, genetic counselors, nurses, oncologists, and surgeons) are a common source of medical support for those who undergo genetic testing, women’s family members, especially their partners, are the most likely source of psychosocial support [14,15]. Indeed, prior work has demonstrated that women’s *BRCA1/2* test-related decisions are often discussed with their partners, and most women feel supported by their partners [16,17]. However, these same data also indicate that, in the face of less support and greater protective buffering in partnered couples (ie, hiding worries, denying concerns, and engaging in avoidant behaviors), poorer psychological outcomes can ensue [10,18,19]. By contrast, greater partner support predicts better psychological outcomes among these dyads [16].

In light of this, it is critical that families facing the risk of hereditary breast and ovarian cancer be adequately supported and empowered, both medically and psychosocially, before and after learning about their disease risk [19-21]. Given the limited time and resources of most cancer care providers to offer ongoing psychosocial and educational support to women tested for *BRCA1/2* mutations and their family members, it is imperative that adjunctive models of psychoeducational support be offered outside of the health care setting to better meet the needs of women who are at risk of familial breast cancer and their partners. Psychoeducation, which is a well-known intervention model for providing informational and psychosocial support for chronically ill women and their partners [22-25], may be an important intervention method for families facing the risk of hereditary breast and ovarian cancer as an adjunct to standard cancer care and cancer prevention.

The Internet is a primary resource for those seeking information and support about cancer [26,27]. Internet-based resources are particularly valuable tools for those facing a risk of cancer and other chronic diseases, as they can provide timely, relevant resources [28-30]. Internet-based resources are also commonly available for persons with a known risk of hereditary breast cancer. For example, the National Cancer Institute, the American Cancer Society, Susan G. Komen for the Cure, and other leading breast cancer advocacy groups sponsor websites devoted to educating the public about hereditary breast cancer, prevention, treatment, and related issues. However, and despite the familial nature of hereditary breast cancer and the involvement of relatives in genetic counseling and testing [16], Internet-based psychoeducation has not been developed specifically for partners of women surviving with hereditary breast cancer and those at risk but who have not developed disease (ie, previvors).

Partners of previvors and survivors, especially male partners, may prefer Internet-based resources to face-to-face psychoeducation because they offer anonymity surrounding sensitive topics and emotional experiences, and provide direct access to needed information [30-33]. It is likely that partners (and male partners in particular) have specific and unique needs for psychoeducation that could assist them in supporting previvors and survivors, which may include education about hereditary breast cancer, helping facilitate decisions about *BRCA1/2* genetic counseling and testing and options after testing, establishing and maintaining open communication within the partnership about hereditary breast cancer and related concerns, and performing supportive behaviors and managing stress and uncertainty in the face of previvors’ and survivors’ hereditary cancer risk and risk of cancer in the family [32,34]. To date, however, there has been no systematic examination of the psychosocial support needs of partners of previvors and survivors of hereditary breast cancer.

To address this gap, we report on the results of an online community needs assessment conducted with this target population. Specifically, the assessment, which was conducted by Facing Our Risk of Cancer Empowered (FORCE), sought to describe the need for psychoeducational interventions that could be offered via the Internet and other communication channels among partners of hereditary breast cancer previvors and survivors. FORCE maintains in-person, telephone, and Web-based programs that provide information, peer support, resources, and a community tailored to individuals at high genetic risk. Its website (www.facingourrisk.org) is the leading site specifically devoted to the community of hereditary breast cancer previvors and survivors. It is expected that the findings of this assessment would be used to inform the planning and development of new interactive and Internet-based psychoeducational interventions for this target population.

## Methods

### Overview

This is a secondary analysis of data originally collected by FORCE through a Web-based survey to determine its online community members’ needs. The work was guided by the PRECEDE portion of the PRECEDE-PROCEED conceptual framework for designing health promotion programs [35,36]. Briefly, the PRECEDE framework refers to Predisposing, Reinforcing, and Enabling factors in educational diagnosis and evaluation [36]. According to PRECEDE, a critical initial step in planning health promotion and intervention programs is to understand gaps between resources that are currently available within the target community and community members’ perceived needs for additional resources [35,36].

### Setting

The Internet survey was conducted by FORCE, Inc. Based in Tampa, FL, USA, FORCE is a national 501(C)3 not-for-profit organization devoted to raising awareness about hereditary breast and ovarian cancer and *BRCA1/2* mutations. The FORCE website is the foremost lay Internet site devoted to the cancer education and support needs of persons with or at risk for hereditary breast cancer and their family members. The FORCE website contains timely and accurate information about hereditary cancer, cancer risk assessment, and other related topics. In addition to educational information, resources offered by the organization include national and local outreach groups, online webinars, print brochures, a toll-free helpline, periodic newsletters, and an annual educational meeting.

### Recruitment and Data Collection

The needs assessment sample consisted of 143 partners who responded to the Web-based survey. The anonymous survey took approximately 10 minutes to complete, and no personally identifying information was collected. The survey was made available via the FORCE website homepage from November 2010 to February 2011. The heading for the survey targeted spouses or partners of women who were at risk for hereditary breast cancer, defined specifically as having *BRCA1/2* genetic mutations or a family history of breast cancer, or who had a *BRCA1/2*-linked cancer. Respondents were asked to affirm the following statement prior to completing the survey:

Participation in this survey is limited to spouses and partners (men and women, age 18 or older) of women with a BRCA mutation or family history of cancer. By continuing, you are agreeing with the above terms and volunteering to participate. If you do not wish to participate, please close your browser or exit at any time.

The protocol for this secondary data analysis was reviewed and approved by the Institutional Review Board at Georgetown University.

### Measures

#### Demographics

The demographic characteristics assessed were respondent age, gender, race/ethnicity, and highest level of education attained. In addition, 2 items assessed whether respondents had any children, and whether they had any female children.

#### Clinical Characteristics

Clinical characteristics assessed included whether respondents’ partners had a diagnosis of breast cancer, had been tested for a *BRCA1/2* genetic mutation, and had surgery to remove her breasts (ie, prophylactic mastectomy) or ovaries (ie, prophylactic oophorectomy), or for breast reconstruction. Based on these items, 3 variables were created to indicate whether each respondent’s partner had (1) a diagnosis of breast cancer, (2) received *BRCA1/2* genetic testing, and (3) undergone any of the 3 surgery types we inquired about.

#### Internet and Email Usage

Because FORCE has a large and active online community, respondents’ use of technology was presumed to be moderately high. However, to evaluate this presumption among partners, Internet use was formally assessed using 2 items. The first item asked “How often do you go online to access the Internet?” with response options ranging on a 4-point Likert-type scale from never to very often (more than once/week). The second item asked “When you go online, where do you primarily access the Internet from?” with response options including home, work, Internet café, family members or friends’ home, and other. Email use was assessed by asking “Do you have an email address for your personal use?” Respondents were dichotomized as high Internet users if they accessed the Internet very often, did so from home, and had an email address for personal use; all other participants were categorized as low Internet users [37].

#### Preferred Psychoeducational Content

We used 7 items to assess respondents’ preferences for psychoeducational content. From the perspective of partners, topics queried included understanding my role/knowing what to expect, communicating with my spouse/partner, helping my spouse/partner make decisions, communicating with adult relatives, communicating with children, intimacy after diagnosis or surgery, and speaking with other spouses/partners going through a similar situation. Response options for each item were yes, no, and I don’t know. Principal components factor analysis of these 7 items confirmed a single-factor solution (eigenvalue = 2.93). Items were analyzed individually and an overall content score was also created by summing responses to the 3 items where yes, I don’t know, and no were again assigned a value of 2, 1, or 0, respectively (range 0–14, Cronbach alpha = .75). As such, higher scores reflected stronger preferences for more psychoeducational content.

#### Communication Channels

We used 11 items to assess respondents’ likelihood of using psychoeducational resources offered through the following communication channels: regular mail, toll-free telephone line, email, national and local FORCE meetings, printed booklet or guide, periodic newsletter, expert teleconference or webinar, video or DVD, Web-based message board, and Web-based chat. Items were preceded by a statement instructing respondents to indicate how likely they would be the find the following resources and information for spouses or partners to be useful. Response options were based on a 7-point Likert-type scale with anchors at values of 1 (not at all likely) and 7 (very likely).

These 11 items were subsequently factor-analyzed to empirically derive subscales with eigenvalues >1: we identified 3 subscales. Subscale scores were then created by averaging responses to the items loading on each subscale to ensure that all subscales were based on a common underlying metric. The *s* *elf-* *help* subscale consisted of 4 items assessing the likelihood of using a printed booklet or guide, newsletter, and materials delivered via postal mail and email (eigenvalue = 4.8, Cronbach alpha = .82, mean 5.1, SD 1.4, range 1–7). The *o* *nline i* *nteraction* subscale consisted of 4 items assessing the likelihood of using resources offered via a Web-based message board or chat, embedded video or DVD, and expert webinar or teleconference (eigenvalue = 1.6, Cronbach alpha = .82, mean 4.2, SD 1.5, range 1–7). Finally, the *i* *nterpersonal i* *nteraction* subscale consisted of 3 items assessing the likelihood of using in-person resources offered via national and local FORCE meetings, as well as a telephone hotline (eigenvalue = 1.1, Cronbach alpha = .76, mean 3.6, SD 1.6, range 1–7).

#### Perceived Need for Psychoeducation

We used 3 items to assess respondents’ perceived need for psychoeducation. The items were introduced to respondents with a brief description of the informational resources that could be made available through FORCE, followed by items assessing whether respondents (1) perceived a *need* for more resources or support, (2) *wanted* more resources or support, and (3) would *use* more resources or support. Response options for each item were yes, no, and I don’t know. An overall score was created by summing responses to the 3 items with assigned values of yes (2), I don’t know (1), and no (0) (range 0–6, Cronbach alpha = .70): higher scores reflect greater need.

### Data Analysis

Statistical analyses were conducted in several steps. First, we used descriptive statistics to characterize the study sample and describe their preferences and perceived needs for psychoeducation. Second, we subjected communication channel subscale scores (ie, self-help, online interaction, and interpersonal interaction) to a hierarchical cluster analysis to determine first-order groupings of respondents based on their self-reported likelihood of using resources offered through specific communication channels. We selected the unweighted pair group method using arithmetic averages, which defines clusters based on the average pairwise proximities between clusters of all pairs of observations [38,39]. This method has performed well under various conditions in Monte Carlo simulation studies and was suitable for the data [39]. The analysis produced 3 clusters of potential users of psychoeducational interventions with eigenvalues >1. To confirm the validity of these groupings, we conducted pairwise comparisons of mean subscale scores for preferred communication channels across potential user clusters, applying the Tukey post hoc adjustment for multiple comparisons [40].

Subsequently, bivariate tests (ie, *F* tests, *t* tests, χ^2^ tests) examined relationships between demographic and clinical characteristics, and perceived need for psychoeducation, across user clusters. Finally, a multivariate analysis of variance (MANOVA) examined variability in need across users, adjusting for participant demographic characteristics (ie, age, gender, race/ethnicity, any children) as covariates [40].

Prior to analyses, we studied patterns of missing data in focal variables, including preferred communication channels, psychoeducational content, and need for support. While data were missing for any one of these variables for only a few participants (ie, ≤10%), Little’s [41] χ^2^ test for data missing completely at random indicated the presence of identifiable patterns of missing data (χ^2^_32_ = 56.7, *P* = .005). To account for missing data on these variables, we used a single regression-based imputation method, imputing predicted values based on demographic characteristics including age, gender, and race/ethnicity. This method has been shown to be adequate for imputing missing values when the proportion of participants with missing data is relatively low, as was the case for our sample [40].

## Results

### Study Sample

Characteristics of the respondent sample (n = 143) are displayed in Table 1. Respondents averaged 45.8 years of age. A majority were male (86.0%), white (94.4%), had a college education or higher (86.7%), and had 1 or more children (69.2%). Most respondents reported that their spouse/partner had *BRCA* *1/2* genetic testing previously (91.6%) and some form of surgery (78.3%). Most respondents (55%) were categorized as high-level Internet users, and their need for psychoeducation was moderately high (mean 4.6, range 0–6). Descriptive information for partners’ preferred psychoeducational content is displayed in Table 2. As shown, there was uniformly strong interest in all content areas presented, and particularly for content focusing on the partner’s role and knowing what to expect, decision making, communication, and intimacy.

**Table 1:** Sample characteristics (n = 143)

**Demographics**			Mean	SD	n	%
	Age (years)		45.8	10.5		
	Gender					
		Male			123	86.0
		Female			20	14
	Race/ethnicity					
		White			135	94.4
		Nonwhite			8	6
	Education					
		< College			19	13
		≥ College			124	86.7
**Family characteristics**						
	≥1 Child				99	69
	0 Children				43	30
	≥1 Female child				73	51
	0 Female children				70	49
**Clinical characteristics**						
	Breast cancer diagnosis				48	34
		No			91	64
	*BRCA* testing				131	91.6
		No			12	8
	Breast/ovarian surgery				112	78.3
		No			31	22
**Internet use**						
	High				78	55
	Low				65	45
**Need for psychoeducation** (range 0–6, alpha = .77)			4.6	1.8		
**Communication channel** ^a^						
	Self-help (range 0–7, alpha = .82)		5.1	1.4		
	Online interaction (range 0–7, alpha = .82)		4.2	1.5		
	Interpersonal (range 0–7, alpha = .76)		3.6	1.6		

^a^ Scores based on the average response to items within each subscale based on a 7-point Likert-type scale with anchors at values for 1 (not at all likely) and 7 (very likely).

**Table 2:** Preferred psychoeducational content

**Topic** ^a^		Mean	SD
	Understanding my role/knowing what to expect	1.89	0.45
	Helping my partner make decisions	1.75	0.63
	Communicating with my spouse/partner	1.68	0.73
	Intimacy after diagnosis/surgery	1.67	0.73
	Speaking with others undergoing a similar experience	1.47	0.82
	Communicating with children	1.45	0.84
	Communicating with adult relatives	1.24	0.90

^a^ Response options for each item were yes, I don’t know, and no and assigned values of 2, 1, or 0, respectively.

### Cluster Analysis

The cluster analysis of communication channels identified 3 distinct groups of partners based on their likelihood of using psychoeducational interventions (Table 3). The smallest proportion of participants (18%, n = 26) fell into the low-use cluster (eigenvalue = 1.36), which was characterized by the lowest likelihood of their expected use of all 3 resource types. A majority of respondents (55%, n = 78) were characterized by the moderate-use cluster (eigenvalue = 4.58), which included intermediate levels of need for each resource type. Finally, just over one-quarter (27%, n = 39) of partners were classified in the high-use group (eigenvalue 1.04), with the highest average likelihood of using all 3 communication channels. All pairwise mean comparisons of the likelihood of using self-help, online, and interpersonal interaction resources were significantly different across psychoeducational intervention use clusters at *P* < .05 using the Tukey post hoc adjustment, confirming cluster validity [38].

**Table 3:** Cluster analysis of partners’ preferred communication channels

		Likelihood of psychoeducational intervention use (clusters)					
		Low (26/143, 18%)		Moderate (78/143, 55%)		High (39/143, 27%)	
**Communication channel^a^**		Mean	SD	Mean	SD	Mean	SD
	Self-help	3.5	1.5	5.2	1.1	5.8	1.2
	Online interaction	2.1	1.1	4.4	1.0	5.3	1.1
	Interpersonal interaction	1.8	0.95	3.1	0.95	5.7	0.70
Eigenvalue		1.36		4.57		1.04	

^a^ Values for communication channel are based on a 7-point Likert-type scale with anchors at values for 1 (not at all likely) and 7 (very likely). All pairwise mean comparisons of preferred communication channels across clusters are statistically significant at *P* < .001 using the Tukey post hoc test, except for high and moderate clusters for the self-help channel, where *P* = .02.

### Bivariate Relationships

Participants in the low-, moderate-, and high-use clusters did not significantly differ based on demographics, family characteristics, clinical characteristics, or Internet use. Significant differences in partners’ perceived need for psychoeducation were evident across clusters. Specifically, those in the moderate-use and high-use clusters reported significantly greater need than did participants in the low-use cluster (*F*_2,142_ = 13.3, *P* < .001).

### Multivariate Analysis of Variance

Findings from the MANOVA indicate that, after adjusting for demographic covariates including age, gender, white race, and any children, significant variability existed in partners’ need for psychoeducation across clusters (Wilks lambda = 0.88, *F*_2,132_ = 9.15, *P* < .001; group main effect *F*_2,138_ = 9.15, *P* < .001). Examination of pairwise comparisons of adjusted mean need across clusters indicated that partners in the low-use cluster (mean 3.8, SE 0.48) reported significantly less need than partners in the high- (mean 5.7, SE 0.41, *P* = .002) and moderate-use (mean 5.2, SE 0.38, *P* < .001) clusters: need did not differ significantly between partners in the high- and medium-use clusters (*P* = .32).

## Discussion

The purpose of this study was to describe and determine the need for psychoeducational interventions among partners of previvors and survivors of hereditary breast cancer, with an emphasis on using the Internet and other remote communication channels to reach a geographically dispersed target population. The findings suggest that partners have a moderately high self-assessed need for psychoeducational interventions, and tend to prefer printed self-help and interactive online resources (though other interpersonal channels received interest as well). With respect to content that would likely resonate with partners, all topics inquired about during the needs assessment received endorsement and particularly topics normalizing the partner’s role as an informed supporter of women facing hereditary cancer risk, with training in coping and communication skills. Interestingly, we were able to empirically derive 3 distinct groups of potential users of psychoeducational interventions based on their communication channel preferences, including those who may be the most and least likely to use self-help, as well as online and interpersonal interaction-based resources. It is expected that these results can inform planning and development of new interactive, Internet-based intervention tools that are specifically directed toward and designed to meet the psychoeducational needs of previvors’ and high risk survivors’ partners.

Prior studies of the use of Internet-based information and support resources suggest that our findings may, in part, reflect the fact that the needs assessment sample comprised predominantly men. Partners endorsed a high need for psychoeducation, which is consistent with earlier research demonstrating that men are less likely than women to participate in in-person psychosocial cancer support groups, yet express levels of need for resources and support similar to those of women [42,43]. Additionally, the sample of partners indicated a strong need for Internet-based resources, especially partners in the high-need group. Men in particular may be more likely to use Internet-based psychoeducational resources because they allow for anonymous discussion of sensitive topics (eg, intimacy and relationship concerns) [33,44]. Men have also been observed to participate less frequently than women in online discussion forums surrounding cancer, but when they do they tend to express sentiments of fear and anxiety [45] and raise emotional topics, such as the prospect of losing their spouse or partner to cancer [31,32,46]. Moreover, partners of women facing breast cancer experience stress and anxiety when confronting complex issues surrounding breast cancer risk [34,47,48]. Because they themselves may be distressed and in need of information about sensitive topics, partners of breast cancer survivors and previvors may prefer the privacy and anonymity offered by Internet-based psychoeducation for meeting some of their needs.

Our findings also raise important considerations regarding the diversity of partners and the need for psychoeducation targeted toward special populations. While a majority of the sample were men, a significant minority (14%) of partners who responded to the online needs assessment were women. This suggests that when developing resources targeted to partners’ needs, one should take into consideration the diversity within this population, including same-sex couples. Evidence regarding the impact of breast cancer on same-sex couples and their need for psychoeducation is scarce [49-51]. Further research is needed to examine potential unique resource needs of same-sex couples as they face the risk of hereditary breast cancer.

Male breast cancer is rare and accounts for only a small proportion of hereditary breast cancer cases (<1%) [52,53]. Men determined to be *BRCA1/2* mutation carriers have elevated risks for breast and prostate cancer, as well as other forms of the disease [54]. Families of male breast cancer previvors and survivors may face similarly complex issues when confronted with decisions surrounding preventive screening and treatment, and the possibility of mutations in offspring and other first-degree relatives [18,55]. Male breast cancer previvors and survivors, and their partners also likely have unique needs for resources to help them navigate these complex issues, which may necessitate targeted interventions and should be investigated in future studies.

Internet-based resources may be ideally suited to address the psychoeducational needs of partners as described herein. Internet-based tools could enable health professionals to effectively incorporate self-help materials that could be downloaded or printed from the Web, as well as leverage interactive multimedia aids such as videos, expert webinars, and online discussion forums to deliver content, provide support, and engage participants [28]. Moreover, Internet-driven intervention-delivery approaches provide ample flexibility to tailor content to individuals’ needs and offer targeted materials for population subgroups, which are strategies that have been shown to improve intervention outcomes [56]. Finally, an Internet-based format would provide partners with opportunities for social networking and interaction with other partners, and offer cancer care providers the opportunity to participate in the online space. Such components would attend to the needs of partners preferring to experience more interpersonal connections and communications as well.

Professionally facilitated, Internet-based psychoeducational interventions implemented in diverse populations [30], including those with cancer [28], have shown promising results. Intervention platforms such as the Comprehensive Health Enhancement and Support System improve psychosocial outcomes among women with breast cancer [57-59]. There remains, however, a need to rigorously develop and evaluate Internet-based resources with the specific needs of partners in mind. They are not directly affected by hereditary cancer themselves, yet constitute an important group in the comprehensive cancer care-delivery system. The results of this assessment will be useful to inform efforts to reach out to and support their roles in the future.

Moving forward, it is critical to carefully investigate the ways in which Internet-based psychoeducation and support resources can be integrated into the delivery of health care services for hereditary breast cancer, such as *BRCA1/2* genetic counseling and testing services [30,60]. The Internet is a primary resource for those seeking information about cancer [26,27], and health care providers must acknowledge that patients will use the Internet as a source of information whether it is suggested to them or not. Therefore, it compels providers to take responsibility for directing their patients toward credible, evidence-based resources [61]. Clinical providers should also be aware of important considerations surrounding Internet-based information and support resources, such as the lingering digital divide, which may prevent patients of certain sociodemographic backgrounds (eg, lower socioeconomic status, minority racial/ethnic groups, and the elderly) from having equal access [62,63].

### Limitations

The findings reported herein should be interpreted in light of the limitations of this work. The original needs assessment data relied on a convenience sample of partners responding to an Internet-based survey of unknown denominator (reach). Moreover, our focus was on partners of previving and surviving women, and did not include partners of affected men. We caution against generalizing the findings to the broader population of partners. The potential influence of selection bias is also an important consideration, as partners completing the assessment may differ from those who did not. The assessment used self-reported measures of communication channels, perceived need for psychoeducation, and other key constructs. Due to the anonymous, Internet-based nature of the needs assessment, it was also not possible to verify reported clinical characteristics (eg, the partner’s receipt of *BRCA1/2* genetic testing or undergoing surgery).

### Conclusions

These limitations notwithstanding, the findings highlight areas for future research with the goal of developing Internet-based psychoeducational interventions for partners of previvors and survivors of hereditary breast cancer. Though partners have diverse needs, use of self-help materials (either in print or electronic form) and interactive, Internet-based resources seems promising. Additional research is needed to rigorously develop and evaluate Internet-based resources targeting partners. Medical Internet and health communication researchers should work closely with cancer genetic content experts to examine ways in which Internet-based resources can be offered to this target population.

## Abbreviations

FORCE: Facing Our Risk of Cancer Empowered
MANOVA: multivariate analysis of variance
